# TC-Net: Dual coding network of Transformer and CNN for skin lesion segmentation

**DOI:** 10.1371/journal.pone.0277578

**Published:** 2022-11-21

**Authors:** Yuying Dong, Liejun Wang, Yongming Li

**Affiliations:** College of Information Science and Engineering, Xinjiang University, Urumqi, China; Vellore Institute of Technology: VIT University, INDIA

## Abstract

Skin lesion segmentation has become an essential recent direction in machine learning for medical applications. In a deep learning segmentation network, the convolutional neural network (CNN) uses convolution to capture local information for modeling. However, it ignores the relationship between pixels and still can not meet the precise segmentation requirements of some complex low contrast datasets. Transformer performs well in modeling global feature information, but their ability to extract fine-grained local feature patterns is weak. In this work, The dual coding fusion network architecture Transformer and CNN (TC-Net), as an architecture that can more accurately combine local feature information and global feature information, can improve the segmentation performance of skin images. The results of this work demonstrate that the combination of CNN and Transformer brings very significant improvement in global segmentation performance and allows outperformance as compared to the pure single network model. The experimental results and visual analysis of these three datasets quantitatively and qualitatively illustrate the robustness of TC-Net. Compared with Swin UNet, on the ISIC2018 dataset, it has increased by 2.46% in the dice index and about 4% in the JA index. On the ISBI2017 dataset, the dice and JA indices rose by about 4%.

## Introduction

With the rapid development of AI, approaches that integrate AI with the medical field are also flowering everywhere in the medical field. Among them, the combination of medical imaging diagnosis and deep learning is not only a newer branch of intelligent medical diagnosis, but also a hot spot in the digital medical industry. Medical imaging contains massive amounts of data, and even experienced physicians sometimes appear disadvantageous. Artificial diagnosis of medical images requires long-term professional experience and relatively long professional training. At the same time, AI can do more rapidly than expert physicians in both detection efficiency and precision of images, and it can also reduce the false positive rate of human manipulation.

Medical image segmentation occupies a key position in the intelligent diagnosis and analysis of medical images. It plays a vital role in computer-aided clinical diagnostic systems. Its function is to segment essential parts of medical images (such as lesion parts or organ parts) through in-depth learning supervision or unsupervised. To provide a reliable basis and help for doctors in clinical medical diagnosis. With the gradual popularization and application of intelligence, medical image diagnosis also faces the transformation to intelligent medicine. Therefore, improving the accuracy of medical image processing will become an essential direction of the development of medical image processing. Medical image segmentation is an important and challenging stage for clinical medical diagnosis. Common medical image segmentation includes polyp segmentation [1], lesion segmentation [2], cell segmentation [3], etc. This paper mainly studies the segmentation of skin lesions [4].

Because skin lesion images have variable resolution and an uneven proportion of skin lesions included in the images, critical information on the location of skin lesions is difficult to obtain. Skin lesions images are usually rarely directly processed and often require pre-processing of images (cropping, spinning, normalization). Nowadays, it has become an urgent need for AI medical diagnosis to continuously improve the segmentation accuracy of lesion parts in medical diagnosis. But the excessive waste of computer resources can be generated in processing and training, hindering the application of smart medical in real life. Therefore, the research focus of this study is to comprehensively utilize the model’s global and local full-type features to improve the model’s feature extraction ability without preprocessing the datasets.

This paper investigates the application potential of transformer network in dermatological focus segmentation. Interestingly, when this paper is tested on skin lesion datasets using the transformer model, which has achieved significant results so far, The results show that the pure transformer network model can not obtain satisfactory results in the field of skin lesions segmentation. Because during the process of entering dermatological image pictures into the transformer network in the transformer network coding phase, these images were compressed into one-dimensional sequences. The operation of batch sequence processing damages the structure information in the picture, and can not make good use of its complete structure information in the decoding stage. It will eventually lead to a less satisfactory network model segmentation. Inspired by the CNN network, in this paper, we capture the feature context information and spatial feature information of images at various stages through series operations such as convolution. Then try to seek an algorithm that can fuse the local feature information of feature maps and the global feature information of networks.

Therefore, this paper proposes a skin disease segmentation model TC-Net. TC-Net adopts the architecture of Swin Transformer combined with CNN. TC-Net combines Swin Transformer with CNN using a double coding structure. The Swin Transformer branch mainly takes a self-attention approach and adds a sliding window form to acquire feature information. CNN branches operate detailed local information through convolutional series. In this paper, the backbone of the CNN branch network adopts Resnet34 with the pre-training model, and the transformer branch network selects Swin Transform architecture. They work together to obtain the feature information of skin lesions images with different feature degrees. Meanwhile, the structural design of dual encoder fusion enables the model to obtain more extensive feature information. As TC-Net models continue to be profoundly and widely acquired, the perceptual domain of the models also increases. There are some main contribution points as follows:

1. Firstly, a U-shaped network framework TC-Net with a dual encoding structure is designed. TC-Net uses Swin Transformer and CNN as two encoder branches. The combination of the two encoders enables the simultaneous acquisition of global and local information of the input image, while richer feature information is input from the encoder part to the decoder part.
2. Secondly, TC-Net proposes a fusion module of CNN and Transformer, which is used to fuse the local information obtained from the CNN encoding part and the global information obtained from the Transformer encoding part. At the same time, the fused feature information is transformed with the corresponding corresponding patch in order to be input to the Transformer decoder part, and the acquired information features are recovered by up-sampling. The experimental results show that the dual-coding structure improves the performance of the model and the utilisation of feature information at each level.

The following chapters are arranged as follows: the second chapter introduces the relevant research on skin lesions segmentation methods. The third chapter presents the methods proposed in this paper and the experimental settings and parameter settings in detail. The fourth chapter mainly shows the quantitative and qualitative analysis of the methods proposed in this paper and other networks, as well as the ablation analysis of the proposed innovation. Finally, the fifth chapter summarizes and makes a simple arrangement for the future work.

## Related work

This section summarizes the related progress of medical image segmentation in computer vision research.The first part is the convolutional neural network-based medical image segmentation progress, and the second part is transformer-based medical image segmentation progress.

Over the past few decades, the field of computer vision has flourished with the wave of deep learning. Recently, the research on the CNN has not decreased, and transformer architecture has become a new research direction of computer vision. Both of them have good performance in the field of computer vision. Here, we briefly review the traditional segmentation methods based on CNN and the recently proposed Transformer network for segmentation.

### Medical image segmentation based on CNN

Yann et al. Proposed the first standard CNN [5], which is for handwritten character recognition tasks. In the past few decades, many powerful networks have achieved unprecedented success in image segmentation tasks [6]. Alexnet [7] and Vggnet [8] show that increasing the depth of the network by stacking convolution and aggregation layers in the network architecture can obtain rich feature information. Google-Net [9] and Inception-Net [10] proposed to add multiple paths for feature information transmission and proved their effectiveness. Resnet [11] in order to better improve its generalization ability, it is proposed to add fast connections in every two layers of the main network. To optimize the problem of limited acceptance domain in previous studies, some studies regard the attention mechanism as the operator of inter-mode adaptation. Senet [12] and Genet [13] improved the performance of the network by establishing the model of interdependence between channels and adaptively recalibrating the channel characteristic response. The summary is shown in the following table.

U-Net [14] creatively proposed a U-shaped network based on encoding/decoding, which has an irreplaceable position in medical image segmentation. UNet + + [15] designed multi-segment nested and dense jump paths in jump connection to narrow the semantic gap. Attention U-Net [16] enables the model to focus on targets of different shapes and sizes by proposing a new attention gate mechanism. A new structure system is proposed, which uses incomplete and over-complete features to improve the segmentation of small anatomical structures. Double-net [17] adopts the order of two u-nets and adopts spatial cone pool (ASPP) [18]. UNet3+ [19] uses deep monitoring and full-scale skip connection, and combines the mask of the previous era with the feature mapping of the current period in the training process. Abayomi-Alli, et al [20]. propose a new data enhancement technique based on the covariant synthetic minority oversampling technique (SMOTE) to address data scarcity and class imbalance. Kadry, et al [21]. used the VGG SegNet protocol to automate the acquisition of dermatologic lesion sections from digital Dermospy images. The Table 1 shows the related papers and contribution, this table details the contributions of relevant research literature to the field of deep learning.

**Table 1:** The contributions of CNN networks.

Internet	contribution
CNN [5]	The first standard recognition task for handwritten characters
Alexnet [7]	First successful application ReLU[U+3001]Dropout
Vggnet [8]	Increase network depth
Google-Net [9]	Proposed convolutional reaggregation at multiple dimensions
Inception-Net [10]	Add multiple paths for feature information transmission
Resnet [11]	Add fast connections at every two layers of the main network
Senet [12]	Using simple low-level feature aggregation methods
Genet [13]	Aggregates the neuron responses in a given spatial range
U-Net [14]	Creatively proposed a coding/decoding based u-type network
UNet + + [15]	Multiple nested and dense jump paths in the jump connection
Attention U-Net [16]	A new attention gate mechanism is proposed
Double-net [17]	Second-order u-shaped network architecture is used
ASPP [18]	Free multi-scale feature extraction
UNet3+ [19]	Use deep monitoring and full-size jump connections
Abayomi-Alli, et al. [20]	A new data enhancement technique
VGG SegNet [21]	Automated acquisition of dermatological lesion sections

### Medical image segmentation based on transformer

Motivated by the achievement of the transformer [22] in various NLP [23] tasks, with the migration of researchers in different fields, Increasingly transformer-based methodologies are appearing in computer vision tasks. In the current development process of the computer vision field, Vit [24] adopted the network architecture of a pure transformer for the first time and realized the SOTA performance of image recognition by pre-training a large number of datasets. Deit [25] solves the limitation that the transformer needs many datasets in training by introducing an efficient data training strategy and knowledge extraction algorithm. Swin transformer [26] innovatively proposed the powerful mechanism of self-attention based on a mobile window, which has linear computational complexity and refreshes the best results in the fields of image recognition, target detection, and semantic segmentation. It breaks through the limitations of most previous models based on the transformer. Swin transformer adopts a layered architecture, which improves the flexibility of its network architecture. Trans-UNet [27] introduced transformer architecture into the field of medical image segmentation and proved its powerful coding performance. PVT [28] imitates the pyramid structure in CNN and introduces it into Vit to realize various pixel-level intensive prediction tasks by generating multi-scale feature maps. CPVT [29] and CVT [30] are most relevant to our work on conventional transformer groups using convolution projection.Under this line of research, we also try to get better results by investigating different components, the combination of other modules, making up for the deficiencies of the existing transformer, and taking advantage of the current advantages. Although many investigators have successfully applied converters to visual tasks, there are still many aspects that have not shown satisfactory results. Compared with the more established CNNs in the visual field, transformer network architecture still has a lot to develop, especially in acquiring local feature information. Inspired by these methods, we propose an input method combining transformer and CNN. We believe that the unified architecture of transformer-based encoder and decoder can provide robust performance in medical image segmentation.

## Methodology

In this part, we first introduce our research motivation, then describe the overall architecture of the proposed TC-Net network, and finally introduce the bilateral code structure and bilateral code fusion module in detail.

### Motivation

Transformer shows excellent potential in computer vision tasks, which makes this paper explore the solutions based on the transformer. At the same time, the feasibility of applying transformer-based network architecture to dermatological image segmentation tasks is also investigated. In the field of computer vision, it is recognized that the use of transformer network architecture mainly needs to use a large number of data sets or load pre-training models. In view of the small amount of medical image data, the simple transformer network has not made significant progress in the medical field. In the research of skin disease segmentation, the characteristics of CNN convolution operation make CNN unable to correlate and model the global information of the input picture. In recent years, researchers have continuously proposed the information extraction module to strengthen the acquisition of the input picture information by the CNN network, which has been dramatically improved. It is found that the self-attention model in the transformer network model applied in the field of natural language processing can model the global semantic information. Therefore, this paper will use the advantages of CNN to extract local information and transformer to extract global information to reasonably achieve more accurate skin disease segmentation results in the case of limited datasets.

### Network architecture of TC-Net

**Fig 1:**
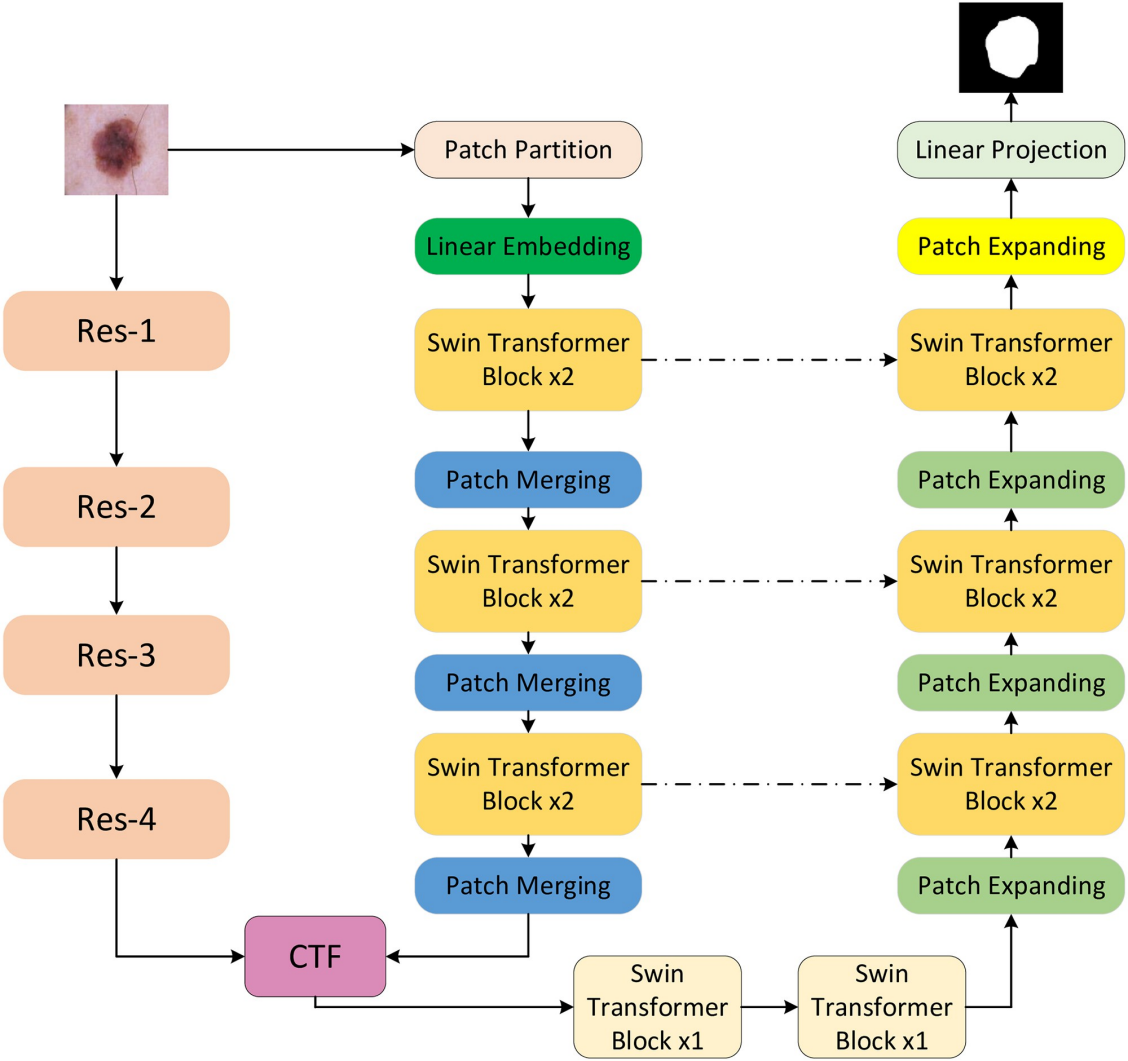
The overall architecture of TC-Net. The image to be processed and input it to the dual coding channel at the same time, then fuse at the bottom of the coding to decode and output the split image.

As shown in Fig 1, in this paper, the double coding structure is adopted in the coding part, the lesion image is input into the two coding structures at the same time, the global feature and local feature are extracted, respectively, and the output results of the coding part are fused and input to the decoding part. In the CNN branch, the image of skin lesions is mainly used to process the whole image to extract feature information. The transformer divides the picture into a series of patch sequences, encodes the position and then inputs it into the transformer branch architecture. The feature is extracted through the Swin transformer module and patch merging structure. TC-Net architecture design the coding output of transformer architecture is adopted to enforce decoding global information of each layer by jumping connection operation, and the captured global information is input to the decoding part. Finally, the global feature information of the output segmentation images is enhanced.

### Double coding structure

The structure of the TC-Net network model proposed in this paper is based on double coding architecture, which is mainly composed of the Resnet module and Swin Transformer module. The coding part on the left is composed of the Resnet module and Swin Transformer module, while the decoding part on the right is only composed of the Swin Transformer module and skip connection module.

**Fig 2:**
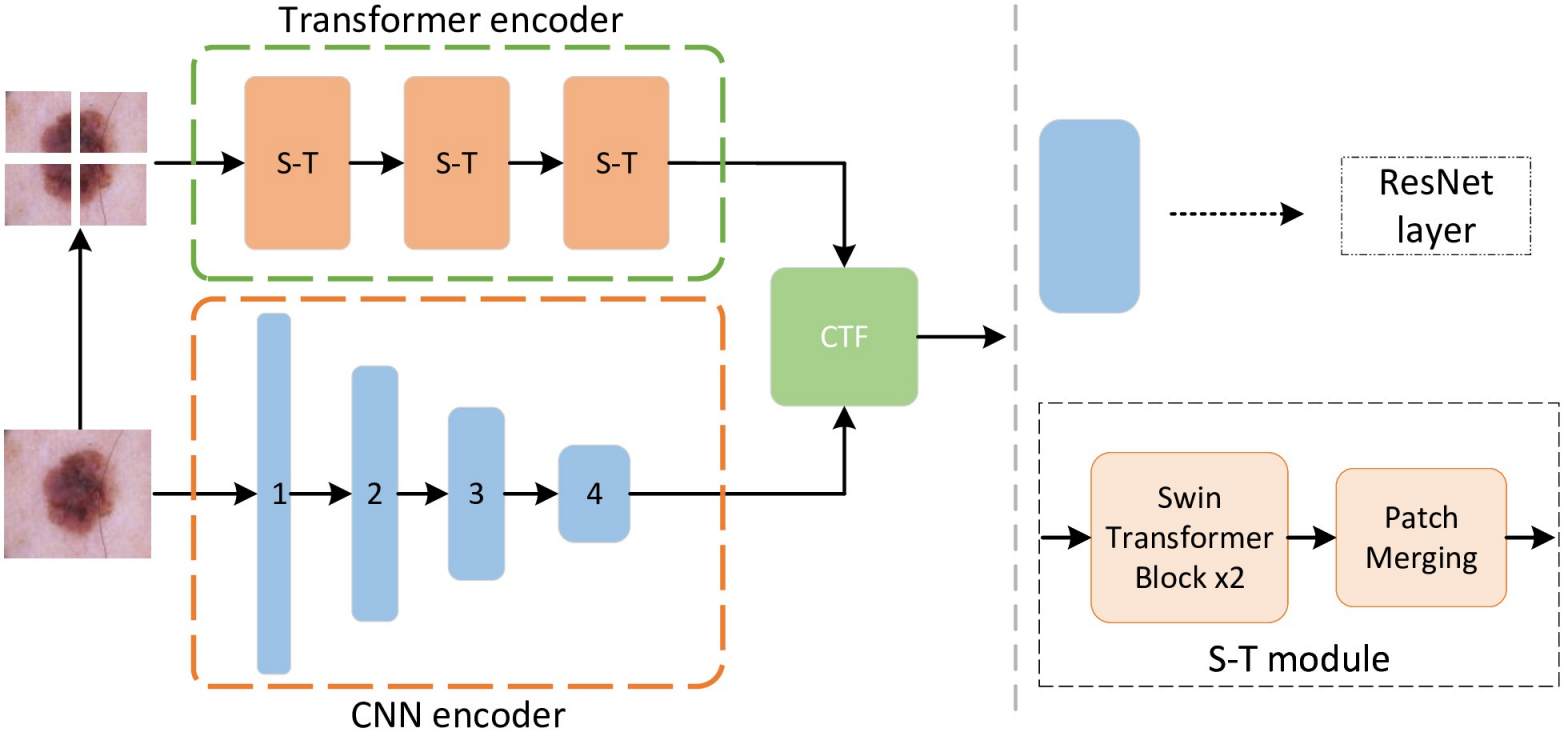
The architecture of feature fusion block.

The framework of its network model is shown in Fig 2. The first coding channel adopts residual structure and convolution structure. By calculating the residual convolution module, rich local feature information is obtained from the input skin disease image. At the same time, the input skin disease image is divided into equal-sized image blocks. The relative position information is added and input into the Swin Transformer module to obtain the global feature information of the input image. The CNN encoder’s local information-dominated feature information output is fused to the global information-dominated feature information output by the transformer encoder via the CTF fusion module.

### The module of CTF

**Fig 3:**
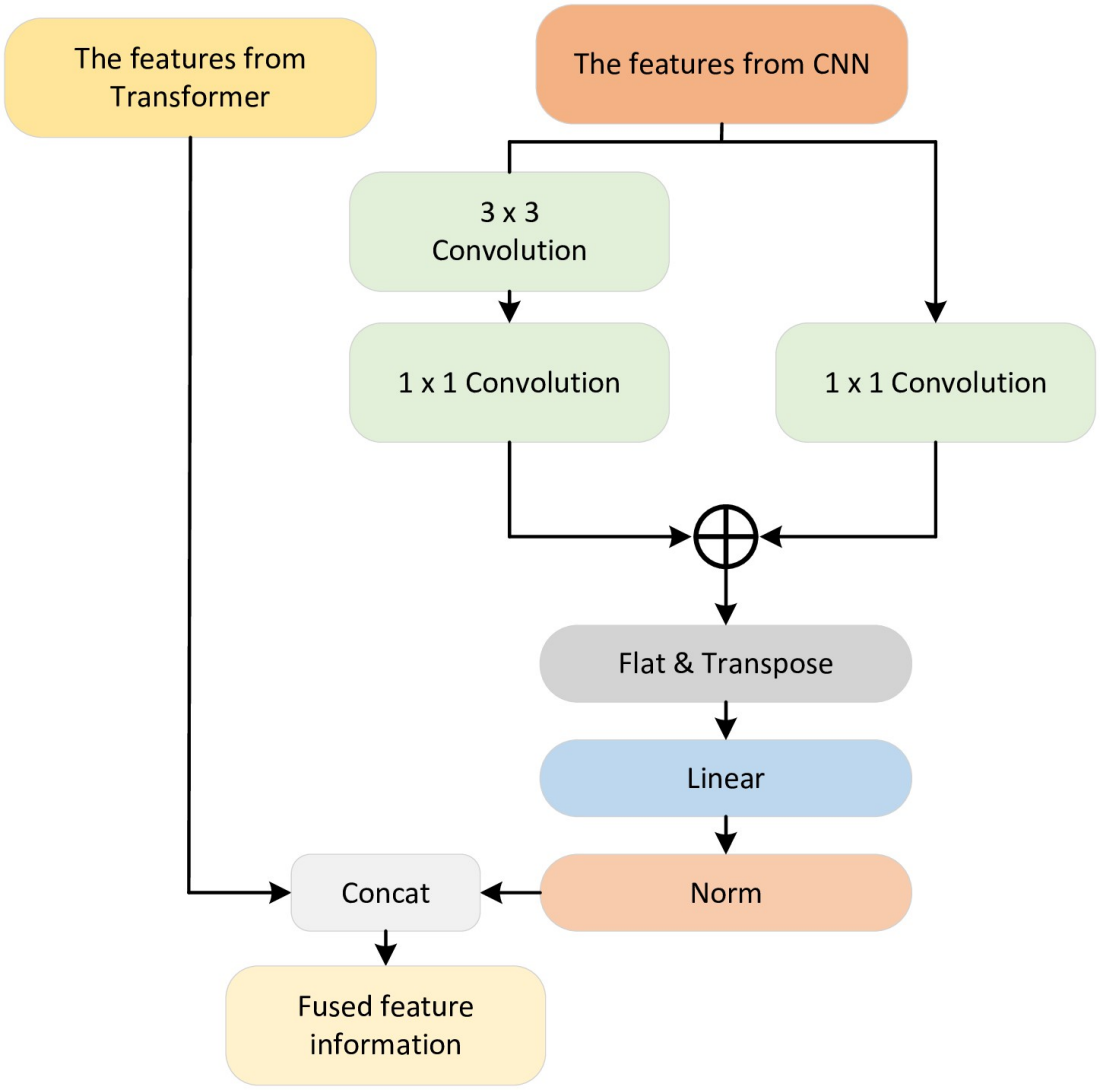
The architecture of CNN and transformer fusion blocks.

Since the transformer and CNN employ different image feature extraction methods, in order to better fuse the different types of feature information extracted by the two encoding parts, this paper proposes a module that combines these two, as shown in Fig 3. Firstly, this paper further obtains the essential information under different receptive fields through two different convolution operations. Then, through a series of operations such as flattening, variable dimension, connection and regularization, the image form processed by CNN is transformed into the same form as that processed by the transformer. Then, the local information is strengthened, summarized, and fused with global information.

The specific operations are as follows: for the CTF module architecture, we have processed two branches according to the picture features from CNN branches. The two branches use the convolution operation of different convolution kernels to obtain various ranges of characteristic information through different sizes of receptive fields. Then, the two are effectively fused and combined with the branch feature information from the transformer through the above operations.

## Datasets and metrics

### The datasets

In the experiment provided in this paper, three famous public skin lesions image datasets ISBI2016 [31], ISBI2017 [32], and ISIC2018 [33] are used to train the network proposed in this paper. These three data sets come from the public data sets of the ISIC challenge competition. Considering the requirements of computer hardware configuration in the natural clinical medical environment, in order to make the algorithm network better applied in real life, this paper does not process the datasets and only adjusts all images and labels to the resolution of 224 × 224 at the same time, In order to test the effect of the network proposed in this paper in the end-to-end natural clinical medical environment,we performed multiple verifications. We selected a total of three datasets, each dataset is divided into three parts: train, valid and test. The Table 2 shows the data distribution of the three datasets, and from the table, we can see that this study divides the three data sets in a certain proportion. So that we can have better robustness in training.

**Table 2:** The introduction of the public datasets.

Dataset	Train	valid	Test
ISBI2016	900	79	300
ISBI2017	2000	150	600
ISIC2018	1815	259	520

### Metrics

To quantitatively evaluate the segmentation performance of TC-Net, we used the following widely recognized evaluation indexes. Accuracy(ACC), sensitivity(SE), specificity(SP), precision (PC), Jaccard index(JA) and dice index(DC) were included. All metrics are closer to 100%, with better segmentation.

## Results and analysis

### Experimental setup

Experimental parameters for this paper were set as follows: for mini-batch training, 12 is the fixed value set for the batch size. The network loss function is the Bce loss function and Dice loss function. The network uses Adam optimizer and Kaiming’s initialization method for optimization and training. The initialization of the network parameters was optimized and trained by Adam optimizer according to the method of the Kaiming et al. The number of iterations of the network is equal to 200, and the initial learning rate is equal to 0.0001, The experiments in this chapter are completed under the Linux system. The deep learning architecture adopted is the PyTorch framework, and the hardware server is NVIDIA Tesla V100.

### Ablation experiment

This paper verifies the effectiveness of the dual encoder network TC-Net and the feasibility and effectiveness of the proposed CNN and transformer fusion module CTF by setting four groups of experiments: CE-Net, Swin-UNet, direct addition of dual encoders, and dual encoder + CTF. This paper is tested on the same dataset (ISIC2018 skin lesions dataset). This paper compares the results of different networks after segmenting the same kind of skin lesions to compare the effectiveness of other modules tested in the ablation experiment. Table 3 shows the performance of the main indicators in each experiment.

**Table 3:** The ablation experiment based on ISIC2018.

Methods	ACC(%)	SE(%)	SP(%)	PC(%)	JA(%)	DC(%)
CE-Net	95.81	88.11	97.88	91.64	81.58	89.71
Swin UNet	95.40	86.81	97.73	90.85	79.62	88.46
Double coding+add	96.03	88.51	98.02	92.07	82.14	90.02
Double coding+CTF	**96.31**	**90.59**	**97.86**	**91.57**	**83.55**	**90.80**

In this paper, four experiments are set as ablation experiments to verify the effectiveness of TC-Net, and the values tested on the dataset are compared through statistical evaluation indexes. It shown in 3, this paper can clearly see that the network method of double encoder addition is better than the traditional pure transformer architecture Swin UNet on the skin disease data set, which reflects the effectiveness of the double coding network structure proposed in this paper. At the same time, it can be clearly observed that the dual encoder with CTF is better than the ordinary dual encoder, which verifies the effectiveness of the CTF proposed in this paper. By comprehensive comparison, the method proposed in this paper is not only superior to the pure transformer architecture Swin-UNet, but also superior to the pure CNN network CE-Net network architecture. Finally, through the evaluation indicators mentioned above, it can be proved that the hybrid dual encoder architecture TC-Net of hybrid CNN and transformer proposed in this paper is effective in skin lesions segmentation.

In the table, the performance of Swin-UNet in each skin disease image segmentation index is lower than that of CE-Net. Compared with Swin-UNet, double coding + add has a corresponding improvement in each index, with an increase of 0.6% on ACC, 2.3% on SE, 1.2% on PC, 2.5% on JA and 1.6% on DC. After joining CTF, the indicators have also improved accordingly.

### Visualization results of ablation experiment

**Fig 4:**
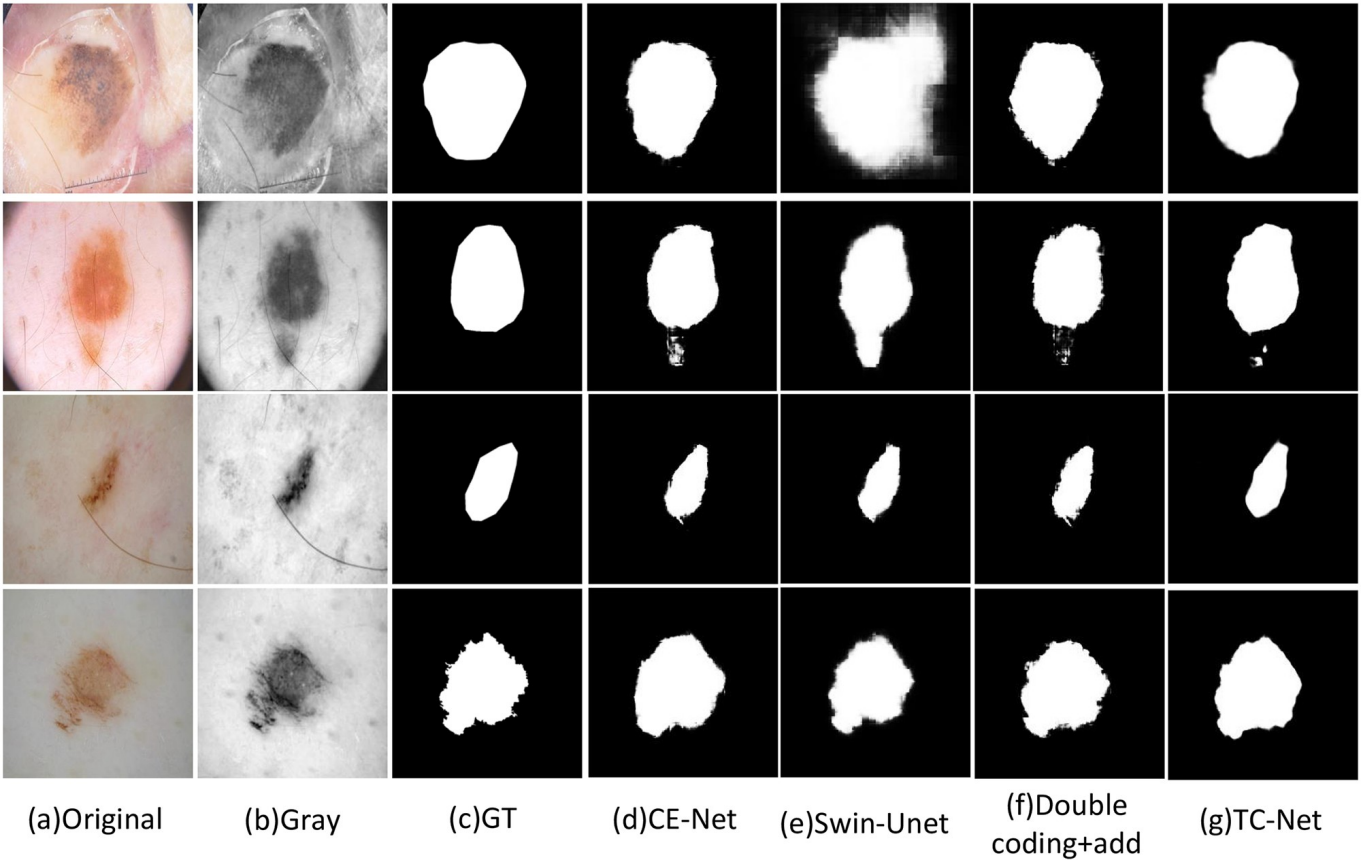
Visual analysis of ablation experiment on ISBI2016. (a) Original. (b) Gray. (c) GT. (d) CE-Net. (e) Swin-Unet. (f) Double coding+add. (g) TC-Net.

**Fig 5:**
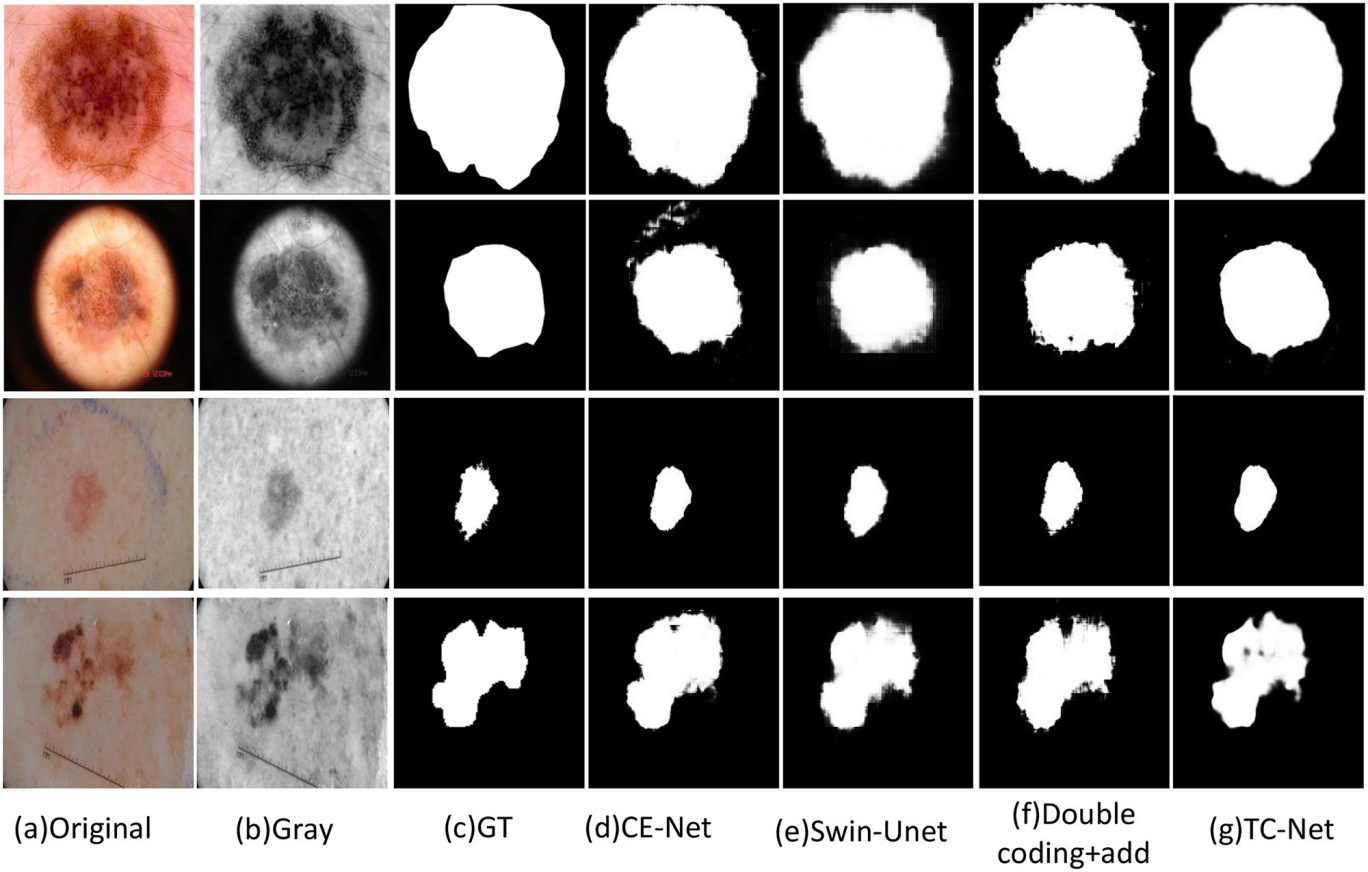
Visual analysis of ablation experiment on ISBI2016. (a) Original. (b) Gray. (c) GT. (d) CE-Net. (e) Swin-Unet. (f) Double coding+add. (g) TC-Net.

In order to illustrate the effectiveness of dual encoder fusion, this paper selects the visual output images of ISIC2018, ISBI2017 and ISBI2016 datasets ablation experiments. This paper verifies the efficacy of the double encoder network TC net proposed in this paper and the feasibility and effectiveness of the proposed CNN transformer fusion module CTF by showing four groups of experiments: CE-Net, Swin-UNet, direct addition of dual encoders, and dual encoder + CTF, This paper compares the results of different networks after segmenting the same kind of skin lesions to compare the effectiveness of other modules tested in the ablation experiment. Figs 4 and 5 show the segmentation effect of skin diseases in four groups of experiments.

As shown in the Figs 4 and 5, the feature information extracted by Swin Transformer performs well globally. After adding the CNN encoder, the local edge information extracted by the network is better. The segmentation image obtained after adding the CTF module is finally appropriate to the label image. In the comparison diagrams of the ablation experiment, this paper can clearly observe the images of the segmentation effect of adding different modules on skin lesions, and intuitively prove the effectiveness of TC-Net and each module.

### Comparative experiment

This paper evaluates TC-Net in the ISBI2016 test dataset, ISBI2017 dataset and ISIC2018 dataset, respectively, and compares the equivalence of ACC, SE, SP, PC, JS and DC, respectively. We compared it with the mature segmentation network, including U-Net, R2U-Net [34], CE-Net [35], SA-UNet [36], UNet3+ and Swin-UNet [37]. Meanwhile, we conducted experiments under identical parameter settings and computational environments to ensure fairness in experimental comparisons. The performance of TC-Net in each index is the best among all networks, which can be proved from Tables 4–6. Compared with the pure transformer network architecture of Swin UNet, and the pure network architecture of CE-Net, TC-net is significantly improved in these three test datasets.

**Table 4:** Comparative experiments based on ISIC2018 dataset.

Methods	Year	ACC(%)	SE(%)	SP(%)	PC(%)	JA(%)	DC(%)
U-Net	2015	94.66	86.03	97.10	88.72	77.43	87.13
R2U-Net	2018	95.09	86.58	97.51	90.00	78.85	88.05
CE-Net	2019	95.81	88.11	97.88	91.64	81.58	89.71
U-Net3+	2020	94.97	85.20	97.77	90.86	78.30	87.71
SA-UNet	2021	94.78	84.87	97.59	90.29	77.63	87.25
Swin UNet	2021	95.40	86.81	97.73	90.85	79.62	88.46
TC-Net		**96.31**	**90.59**	**97.86**	**91.57**	**83.55**	**90.80**

**Table 5:** Comparative experiments based on ISBI2017 dataset.

Methods	Year	ACC(%)	SE(%)	SP(%)	PC(%)	JA(%)	DC(%)
U-Net	2015	92.21	74.38	97.58	89.58	68.30	80.70
R2U-Net	2018	92.28	75.37	97.45	89.38	69.04	81.17
CE-Net	2019	93.49	80.51	97.33	89.92	73.83	84.55
U-Net3+	2020	92.08	72.95	**97.87**	90.69	67.79	80.29
SA-UNet	2021	92.08	76.93	96.66	86.74	68.76	81.06
Swin UNet	2021	92.26	79.01	96.30	86.23	69.86	81.95
TC-Net		**93.68**	**81.45**	97.79	**91.38**	**74.55**	**85.20**

**Table 6:** Comparative experiments based on ISBI2016 dataset.

Methods	Year	ACC(%)	SE(%)	SP(%)	PC(%)	JA(%)	DC(%)
U-Net	2015	94.69	91.30	96.01	89.32	82.18	90.12
R2U-Net	2018	94.43	87.68	97.06	91.49	80.95	89.38
CE-Net	2019	95.94	92.80	97.10	92.06	85.85	92.31
U-Net3+	2020	94.94	90.26	96.74	91.12	82.87	90.54
SA-UNet	2021	94.11	89.46	95.90	88.82	80.14	88.82
Swin UNet	2021	95.02	91.87	96.21	90.39	83.70	91.01
TC-Net		**96.06**	**93.17**	**97.12**	**92.62**	**86.68**	**92.82**

In the ISIC2018 dataset, by careful comparison with Table 4, it can be concluded that compared with U-Net network architecture, TC-Net has increased by 1.4% on ACC, 7% on SE, 0.2% on SP, 1.8% on PC, 6.2% on JA and 4.5% on DC. Compared with the Swin-UNet, the network of TC-Net has increased by 0.99% in the ACC index, about 3.7% in the SE index, about 0.7% in the PC index, about 4% in JA index and 2.4% in DC index. Then compared with the CE-Net network, the network proposed in this paper has increased by 0.5% in the ACC index, about 2.4% in the SE index, and about 2% in the JA index and 1.1% in DC index.

In the ISBI2017 dataset, by careful comparison with Table 5, Compared with U-Net network architecture, TC-net has increased by 1.7% on ACC, 4.5% on SE, 0.7% on SP, 2.8% on PC, 6.1% on JA and 3.7% on DC. Compared with the Swin-UNet, tthe network of TC-Net has increased by 1.4% in ACC index, about 2.4% in SE index, about 5.1% in PC index, about 4.7% in JA index and 3.3% in DC index. On the ISBI2017 dataset, compared with the CE-Net network, the network proposed in this paper has increased by 0.2% in the ACC index, about 1% in the SE index, about 1.5% in the PC index, about 0.8% in JA index and 0.8% in DC index.

Similarly, in the ISBI2016 dataset, by careful comparison with Table 6, it can be concluded that compared with U-Net network architecture, TC-Net has increased by 1.4% on ACC, 1.8% on SE, 1.1% on SP, 3.3% on PC, 4.5% on JA and 2.7% on DC. Compared with the Swin-UNet network, the network of TC-Net has increased by 1% in the ACC index, about 1.3% in SE index, about 2.3% in PC index, about 3% in JA index and 1.7% in DC index. On the ISBI2016 dataset, compared with the CE-Net network, the network proposed in this paper has increased by 1% in the JA index and 0.5% in the DC index.

In order to better verify the effectiveness of the proposed method, we directly compare it with State-of-the-Art Methods. As shown in Table 7, in the absence of data enhancement,the method we proposed still has corresponding improvement compared with other networks.

**Table 7:** Comparative experiences with state-of-the-art methods on fused networks.

Methods	Year	ACC(%)	SP(%)	JA(%)	DC(%)
MedT [38]	2021	-	-	77.8	85.9
TransUNet [39]	2021	-	-	82.2	89.4
MCTans [40]	2021	-	-	-	90.3
R.Ali et.al [41]	2022	95.4	97.1	-	-
H.Wu et.al [42]	2022	95.2	97	-	-
TC-Net	-	**96.31**	**97.86**	**83.55**	**90.80**

The above contents mainly show and analyze the results of TC-Net and the comparative experiments taken in this paper. Through the above introduction, we can easily understand the effectiveness and universality of TC-Net in the task of skin lesions segmentation. At the same time, it also proves the practical significance of this work in the segmentation of skin lesions.

### The visualization results of the comparison algorithm

In order to illustrate the effectiveness of the TC-Net algorithm and other network structures in the task of skin disease segmentation, this paper selects some segmentation results from three datasets: ISBI2016, ISBI2017 and ISIC2018, to display and explain. As shown in the visualization results, although the location of skin diseases presents different sizes and shapes. The TC-Net architecture integrated by CNN and transformer is better than the pure CNN network architecture and the pure transformer network architecture.

**Fig 6:**
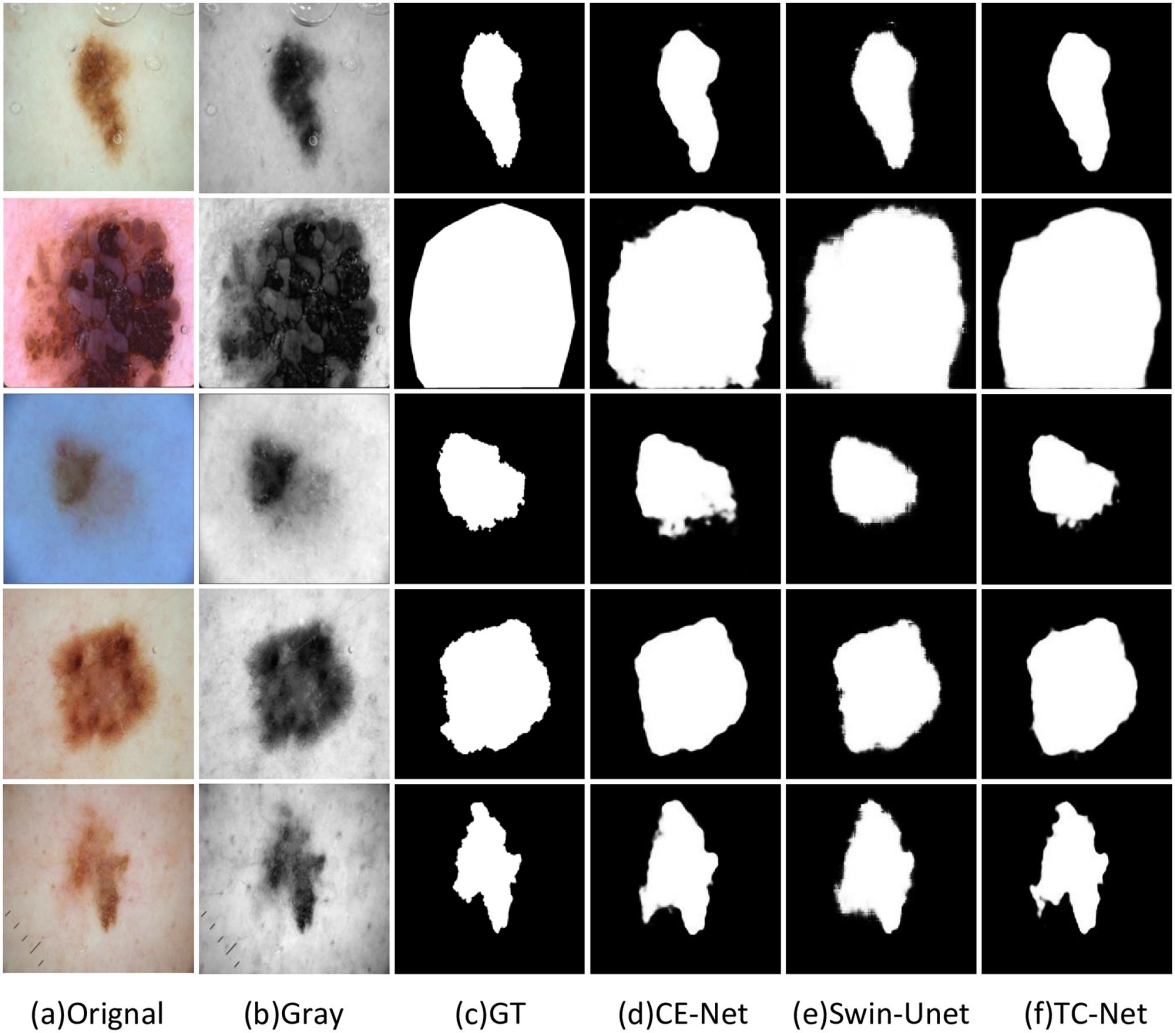
The example on ISIC2018 dataset, (a) original image; (b) Gray image; (c) GT label image; (d) Segmentation image of CE-Net; (e) Segmentation image of Swin-UNet; (f) Segmentation image of TC-Net.

**Fig 7:**
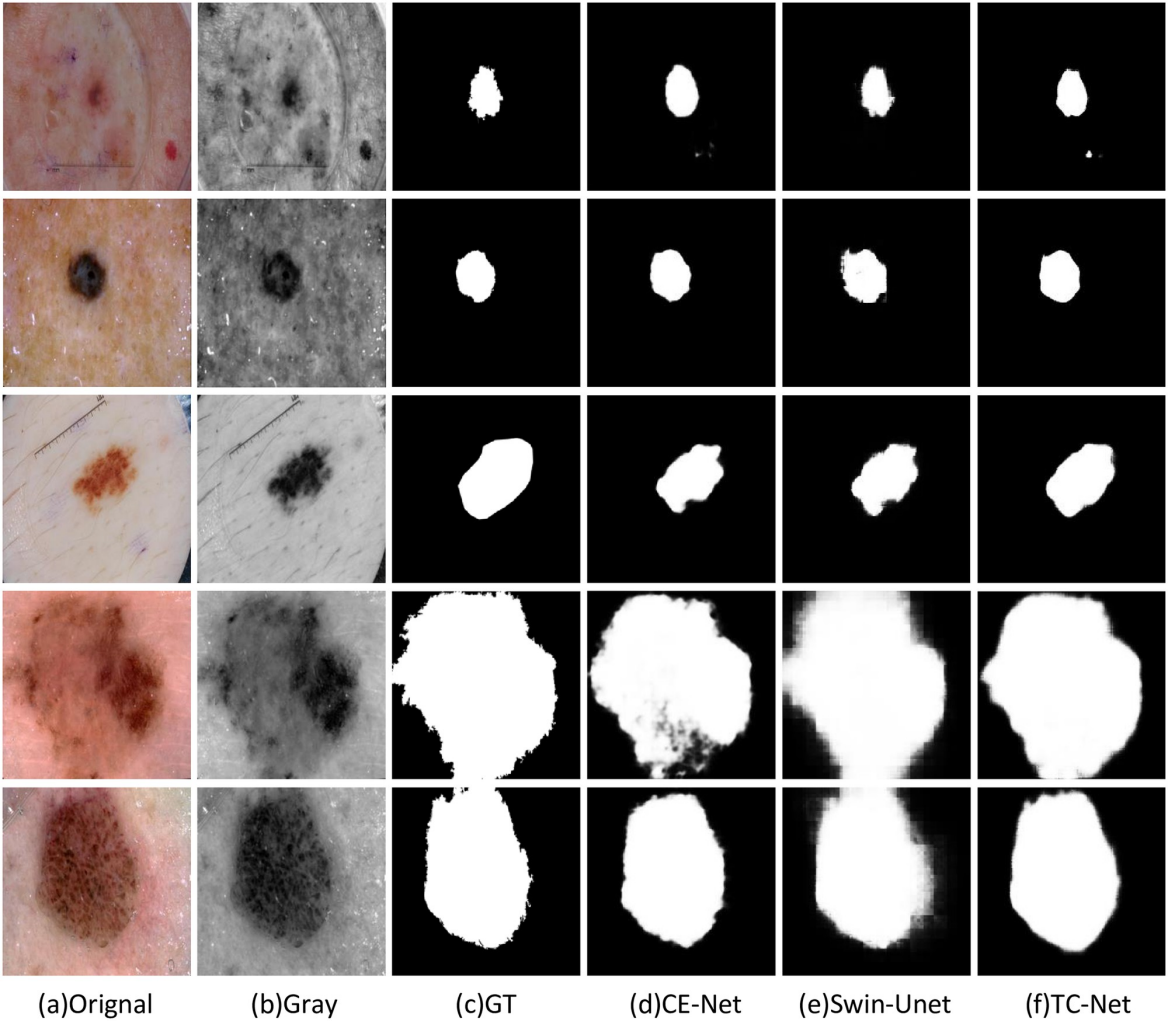
The example on ISIC2018 dataset, (a) original image; (b) Gray image; (c) GT label image; (d) Segmentation image of CE-Net; (e) Segmentation image of Swin-UNet; (f) Segmentation image of TC-Net.

As shown in the Figs 6 and 7, the visual image of the CE-Net does not perform well in the overall connection of the overall edge part, and there is a large difference between the edge and the label image. On the contrary, the Swin-UNet is more in line with the real label value at the segmented edge, but the local situation at the edge is fuzzy. TC-Net combines the advantages of the two networks to achieve the segmentation result more in line with the label value.

**Fig 8:**
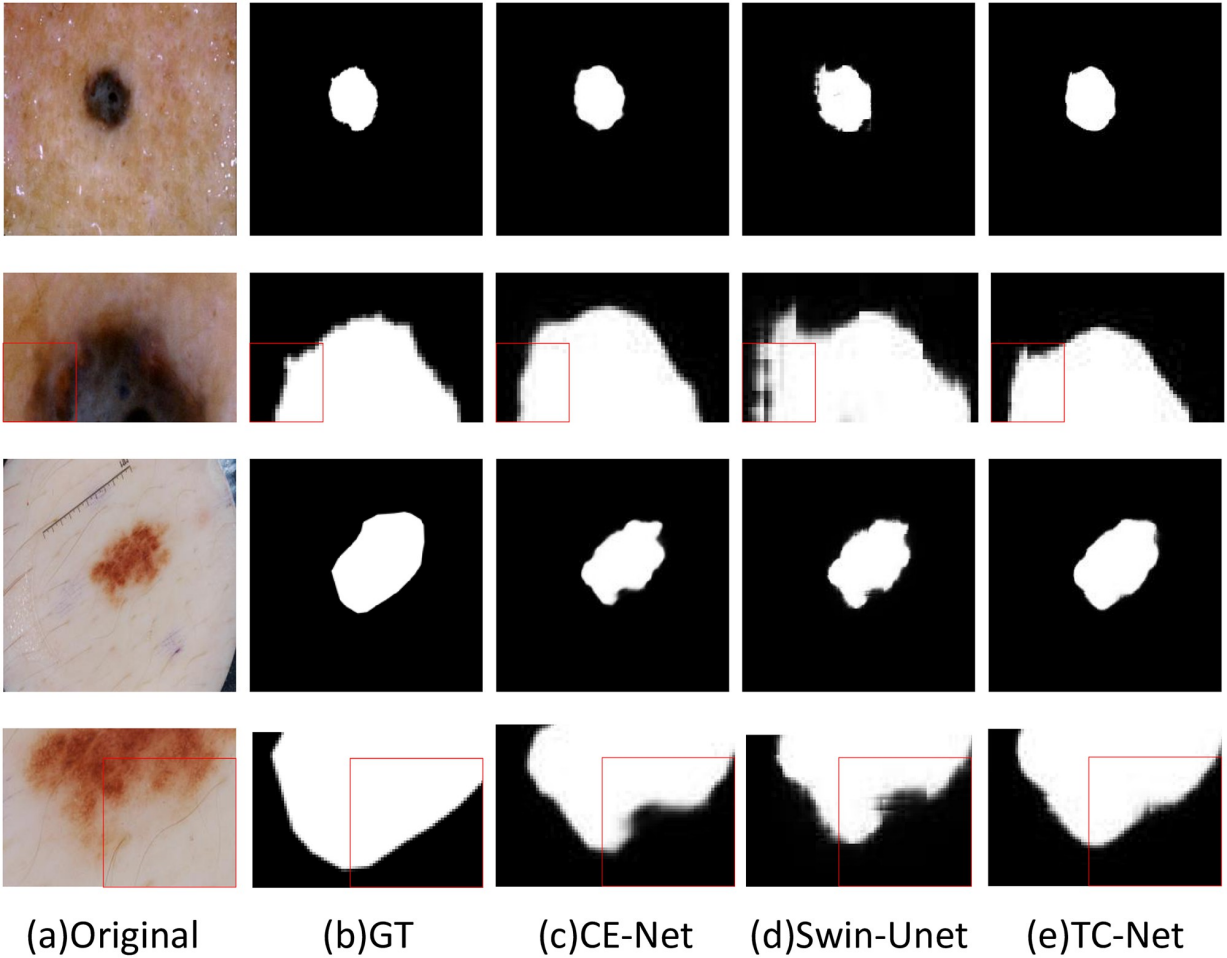
The detail analysis on ISBI2017 dataset, (a) original image; (b) GT label image; (c) Segmentation image of CE-Net; (d) Segmentation image of Swin-UNet; (e) Segmentation image of TC-Net.The red box indicates the segmentation edge information at the same position.

As shown in Fig 8, we pick out two dermatological pictures and their segmentation maps under different networks for more exhaustive analysis, from left to right are respectively the original diagram, the label map, the segmentation map of CE-Net, the segmentation map of Swin UNet and the segmentation map of TC-Net. The excellent performance of the dual coding fusion network TC-Net for skin disease segmentation was excellently demonstrated in terms of the margins of the segmentation map and the fit to the GT map.

To analyze the detailed performance of TC-Net on the skin disease segmentation map, we analyze its performance on the segmentation map in more detail. As shown in the Fig 8, we mark the subtle segmentation edges with a red box. From the mark, we can find that TC-Net can also obtain the characteristic information of the lesion in modest places to get a more realistic partition map.

## Conclusion

Nowadays, in the convolutional neural network, it is mainly through adding innovative feature extraction modules to enrich the critical information in the convolutional neural network. In the transformer network, the advantage of extracting global feature information in the network has also attracted a large number of researchers to explore the field of computer vision. However, the effect of skin disease segmentation using the transformer network alone is not good. In the face of complex and diverse feature information of skin disease focus pictures, this paper proposes a dual encoder segmentation algorithm TC-Net mixed with CNN and transformer. TC-Net is mainly composed of the Resnet module and Swin Transformer module. The coding part on the left is composed of the Resnet module and Swin Transformer module. Local information and global information are convoluted with a transformer fusion module CTF to generate coded output information with rich global information and local information. We selected three publicly available datasets for test validation and performed statistical analysis of the experimental results. Compared to Swin-UNet, it increased the dice index by 2.46% and the JA index by approximately 4% on the ISIC2018 dataset. On the ISBI2017 dataset, both the dice and JA indices increased by approximately 4%. The statistical results show that the proposed network has excellent segmentation performance. However, the current method proposed in this paper only achieves a simple fusion of the two methods, Transformer and CNN, in terms of acquiring information features, and to some extent optimises the segmentation performance of the network TC-Net in segmenting dermatological lesion areas, but the network has no significant advantages in terms of operational speed and network complexity. The next task is therefore to perform a simpler and more effective fusion of network features within the two networks, with some optimisation not only in terms of segmentation effectiveness, but also in terms of overall network performance.
